# Fluctuations of serum cortisol, insulin and non-esterified fatty acid concentrations in growing ewes over the year

**DOI:** 10.1186/2046-0481-67-22

**Published:** 2014-10-18

**Authors:** Tomaz Snoj, Zlatko Jenko, Nina Cebulj-Kadunc

**Affiliations:** 1 Institute of Physiology, Pharmacology and Toxicology, University of Ljubljana, Veterinary faculty, Gerbiceva 60, Ljubljana, Slovenia; 2 Gubceva 7, Ilirska Bistrica, Slovenia

**Keywords:** Jezersko-Solchava breed, Bovec breed, Istrian breed, Growing ewes, Energy metabolism, Cortisol, Insulin, NEFA

## Abstract

**Background:**

The physiological levels of endocrine and metabolic parameters in Slovene autochthonous breeds of sheep are not yet well known, nor are the mechanisms of their adaptability and responses to climate and environmental factors.

Therefore, the aim of this study was to evaluate fluctuations of cortisol, insulin and non-esterified fatty acids (NEFA) in growing ewes over an one-year period. Blood samples were collected monthly from 10 yearling Jezersko-Solchava, 10 Bovec and 10 Istrian ewes. Serum cortisol, insulin and NEFA were measured with commercial kits.

**Results:**

Mean monthly cortisol values fluctuated with low levels in summer and high levels in autumn. Significant peaked cortisol values of 25.69 ± 6.89, 14.67 ± 2.43 and 21.11 ± 7.25 μg/L in Jezersko-Solchava, Bovec and Istrian breed, respectively, were found in September (Bovec breed) and October (Jezersko-Solchava and Istrian breed). Mean monthly insulin values increased during the observation period. The highest levels of 14.60 ± 3.15, 16.03 ± 5.35 and 12.56 ± 2.52 μIU/mL in Jezersko-Solchava, Bovec and Istrian breed, respectively, were observed in the last sample collection in May. NEFA concentrations were found to be low except in some autumn and spring months. The peak values were observed in March for Jezersko-Solchava and Istrian breed (0.60 ± 0.05 and 0.66 ± 0.10 mmol/L), and in April for Bovec breed (0.71 ± 0.11 mmol/L).

**Conclusions:**

Monthly fluctuations of cortisol, insulin and NEFA were measured in all observed sheep breeds, but between-breed differences in monthly values of examined parameters were insignificant. Significantly increased serum cortisol levels were found in autumn for all breeds and were probably associated with the onset of puberty and low environmental temperature. A gradual increase of insulin level in the examined ewes was in parallel with their growth. Significantly higher NEFA values in spring suggest qualitatively insufficient feed supply during that period.

## Background

Autochthonous Slovene breeds of sheep (Jezersko-Solchava, Bovec and Istrian breed) have been bred in the territory of the Republic of Slovenia for several centuries and used for wool as well as meat (Jezersko-Solchava breed) or milk (Bovec and Istrian breed) production. The physiological levels of endocrine and metabolic parameters in these sheep breeds are not yet well known, nor are the mechanisms of their adaptability and responses to climate and environmental factors. In addition to new findings of the physiological characteristics in the autochthonous sheep breeds, their conservation and reintroduction, the knowledge of their properties contributes to the identification of their nutritional status, prevention of metabolic disturbances and economic viability of livestock operations. Such studies also offer reference data for further research of food restriction, long term feeding rhythms and accompanying changes of energy metabolism and metabolical hormones in sheep as models in research of obesity or diabetes in humans. The present study focuses on circulating profiles of cortisol and insulin, which are hormones known to influence energy metabolism, and non-esterified fatty acids (NEFA) levels as a reflection of energy balance in ruminants, in an one-year period.

Cortisol, the most important glucocorticoid in sheep, exerts profound effects on the intermediary metabolism of carbohydrates, proteins and fats, accelerating gluconeogenesis and lipolysis with the release of glycerol and free fatty acids (an insulin antagonistic effect), and inhibiting protein synthesis [1,2]. Cortisol synthesis is regulated through the hypothalamo-pituitary-suprarenal axis [1], but circadian and seasonal fluctuations of this hormone have been described in sheep as having the highest values, particularly in non-pregnant females, during winter or spring [3-6]. Synthesis and secretion of cortisol is accelerated by various forms of stress, like relocation of sheep to a foreign environment or isolation from the flock as well as several physical (ambient temperature), metabolic, immunological and psychological stressors like the time of feeding and handling of animals [7-11].

In contrast to cortisol, insulin promotes fuel storage in the liver by the stimulation of glycogen synthesis and storage as well as the formation of precursors of fatty acid synthesis [2]. The insulin level is strongly affected by feed supply or fasting [1], and is found to be in inverse relationship with NEFA [12]. Additionally, an increase of insulin level was found during animal growth [13]. In sheep, some differences in insulin secretion were described in relation to photoperiod [14].

NEFA as a product of hydrolysis of triglycerides, stored in adipose tissue, can be used as an energy source by several tissues. In ruminants, NEFA levels reflect triglyceride metabolism, since the levels increase during starvation or poor feed supply due to energy demand induced lipolysis [15-17].

In the light of the regulation of energy metabolism and adaptability to environmental conditions, the goal of our study was to follow monthly fluctuations of cortisol, insulin and NEFA in growing ewes of three autochthonous Slovene breeds of sheep over an one year period. Peak cortisol values were expected during the winter period, characterized by extremely low environmental temperatures. With regard to the literature data, seasonal fluctuations were also assumed in insulin and NEFA concentrations. Due to various biological characteristics, use and origin of examined breeds, differences between the breeds for measured values and/or their timeline were also expected.

## Methods

### Animals

The study was performed in a flock of 30 yearling ewes of autochthonous Slovene breeds (10 Jezersko-Solchava, 10 Bovec and 10 Istrian ewes), aged approximately 3 months at the onset of the study (92 ± 8 days in Jezersko-Solchava, 99 ± 16 days in Bovec and 107 ± 11 days in Istrian breed). The ewes were kept on a free pasture of dry Mediterranean karst grasslands utilising a rotational grazing system from the beginning of May to mid-October, separately from the parent flock. During the rest of the year, they were kept free in a stable. They had no contact with rams and were not pregnant at the time of observation. While stabled, they were fed twice a day with hay originating from the farm and containing 5.6% crude protein, 34.2% crude fibre and 1.5% crude fat. Additional rations of a feed mixture for ecological farming (Alpenkorn Lämmer; Unser Lagerhaus, Klagenfurt, Austria) were offered in an average daily amount of 150 g per animal while vitamin-mineral supplementary feed and water were available *ad libitum*.

With regard to the clinical examinations and herd history, the studied sheep were deemed clinically healthy throughout the study and dewormed regularly with albendazol (Monil® 5% suspension, treated orally 0.1 mL/kg; produced by Genera, Kalinovica, Croatia).

Animals were kept on the Karst territory, a sub-Mediterranean region at the latitude 45°41’ N, 14°01’ E and at 760 m above the sea level. Average monthly temperatures during the observational period ranged from 3.7°C in December and January to 22.4°C in July. Daily temperatures below 0°C were observed in the period from October to March. Average monthly rainfall ranged from 56.5 mm in January to 201,5 mm in October [18].

### Sampling

The study protocol and sampling procedure were approved by the Veterinary Administration of the Republic of Slovenia (license number 323-02-201/2003-3).

Blood samples were collected twice monthly with a 10-day interval (on the first Tuesday and the second Thursday of the month) starting in June and finishing in May the following year, at the same time of day: between 8.00 and 9.30 am. Blood was sampled with a jugular vein puncture, using plain 5 mL vacuum tubes (Vacutainer, Becton Dickinson, Heidelberg, Germany). After the samples arrived at the laboratory (4 to 5 hours after sampling), the tubes were immediately centrifuged and the blood serum was stored in aliquots of 300 μL on -80°C until analysed.

Along with blood sampling, all of the animals were also weighed (balance Tru-Test ser. 700, Auckland, New Zealand). The ewes were familiar with humans and used to various handling procedures.

### Measurements of cortisol, insulin, NEFA and progesterone concentrations

Cortisol and insulin concentrations were both determined in the first monthly samples with commercial EIA kits (Diagnostic Systems Laboratories, Webster, USA) following the instructions for users. As reported in the instructions for use, the sensitivity of the cortisol EIA kit is 0.1 μg/L; in the serial dilution studies, the results were linear to the concentrations of 1.6 μg/L to 1.7 μg/L with 81% to 118% recoveries. The precision of the kit was estimated with intra- and inter-assay coefficients of variation (CV), which were 8.77% and 9.91% for low (3.88 ± 0.34 μg/L) and 4.15% and 8.49% for high cortisol values (19.56 ± 0.81 μg/L) in ovine serum. The sensitivity of the insulin EIA kit, reported in the instructions for use, is 0.26 μIU/mL, and in the serial dilution studies, results were linear to the concentrations of 3.2 to 5.6 μIU/mL with 82% to 119% recoveries. The precision of the kit, estimated with intra- and inter-assay CVs, was 8.07% and 5.49% for low (11.31 ± 0.96 μIU/mL) and 9.60% and 9.17% for high insulin values (28.69 ± 2.75 μIU/mL) in ovine serum.

NEFA were measured in the first monthly samples with an enzyme-colorimetric method using a commercial kit (Wako Chemicals, Neuss, Germany) following the instructions for users. The precision of the NEFA assay, as estimated by CVs, was 4.55% for low (0.044 ± 0.002 mmol/L), and 0.86% for high (2.81 ± 0.018 mmol/L) bovine concentrations. The accuracy of the method, established with the analyses of regression, displayed the regression line between the measured and added quantity of NEFA of y = 0.96x + 0.022. The detailed validation of NEFA measurement is described elsewhere [19]. Absorbance was measured with a Cobas Mira biochemical analyser (Hoffman-La Roche, Basel, Switzerland).

Serum progesterone concentrations were measured twice monthly by a commercial EIA kit (Ovucheck® Plasma or Serum Progesterone, Biovet Canada, Saint Hyacinthe, Quebec, Canada). The range of the test was from 1.59 to 95.39 nmol/L of serum. Coefficients of variation were 7.9% for low values (4.88 ± 0.33 nmol/L) and 9.65% for high values (86.38 ± 2,25 nmol/L).

Ovarian activity was evaluated on the basis of progesterone concentrations measured in the both monthly samples. The interval between samplings was selected considering a mean cycle duration of 14–21 days and mean progesterone fluctuations during the oestrous cycle of the ewe. Progesterone concentrations higher than 6.4 nmol/L were considered as indicators for the luteal phase and concentrations below 3.4 nmol/L for follicular phase or anoestrus [20-23]. Two consecutive high progesterone concentrations or one high and one low concentration in the same month were considered as indicators of oestrous cycle and two consecutive low concentrations in the same month as an indicator of anoestrus, as reported previously [22].

### Statistical evaluation

Data was analysed using SPSS 20.0 IBM commercial software (Chicago, USA) General Linear Model with Multivariate Analysis was used for testing of breed × time interaction and one way repeated measures analysis of variance (RM ANOVA) for comparison of values by month for each breed and between breeds for each month. Normality was assessed with a Shapiro-Wilk test and significance with all pairwise multiple comparison procedures (Tukey test). Pearson product moment correlation was used for examining correlations between the results. The results were evaluated as statistically significant at P <0.05 and are presented here as the mean ± standard error of the mean (mean ± s.e.m.).

## Results

### Body weight

The mean body weights of the Jezersko-Solchava, Bovec and Istrian ewes at each weighing time are presented in Table 1. Mean body weight increased with every consecutive weighing except in Jezersko-Solchava ewes where an insignificant loss of mean body weight was observed between April and May.

**Table 1:** **Mean body weight** (**BW**) **of Jezersko**-**Solchava** (**JS**), **Bovec** (**BO**) **and Istrian** (**IP**) **sheep at sampling time**

**Breed**	**BW(kg)**	**Time of weighting(months of the year)**											
		**Jun**	**Jul**	**Aug**	**Sep**	**Oct**	**Nov**	**Dec**	**Jan**	**Feb**	**Mar**	**Apr**	**May**
JS	mean	24.8	28.7	29.6	31.5	33.1	34.9	37.2	37.7	38.5	40.4	40.5	39.9
	s.e.m.	2.71	3.30	2.47	2.49	2.20	2.35	3.56	3.18	2.82	3.07	2.89	2.13
BO	mean	21.0	25.5	25.9	27.7	28.8	30.1	32.5	32.9	34.3	36.4	36.9	37.2
	s.e.m.	3.29	3.69	2.27	3.10	3.50	3.13	3.66	3.97	3.68	4.84	4.67	5.06
IP	mean	23.3	25.2	25.3	27.7	29.2	30.3	31.7	32.0	32.4	33.5	34.4	34.7
	s.e.m.	3.06	3.52	2.63	3.39	3.42	3.86	3.27	3.35	3.42	4.28	3.73	4.42

### Reproductive activity

The number and proportion of ewes within each breed exhibiting signs of ovarian activity are presented in Table 2.

**Table 2:** **The number and proportion of Jezersko**-**Solchava** (**JS**), **Bovec** (**BO**) **and Istrian** (**IP**) **ewes exhibiting signs of oestrus**

**Breed**	**Value**	**Time of the year[months of the year]**											
		**Jun**	**Jul**	**Aug**	**Sep**	**Oct**	**Nov**	**Dec**	**Jan**	**Feb**	**Mar**	**Apr**	**May**
JS	Number [n]	0	0	0	1	4	8	8	7	4	1	0	0
	Proportion [%]	0	0	0	10	40	80	80	70	40	10	0	0
BO	Number [n]	0	0	0	3	4	8	10	9	7	3	2	1
	Proportion [%]	0	0	0	30	40	80	100	90	70	30	20	10
IP	Number [n]	0	0	0	2	1	1	1	0	0	0	0	0
	Proportion [%]	0	0	0	20	10	10	10	0	0	0	0	0

Ovarian activity in all three examined breeds started in September and lasted till March in Jezersko-Solchava breed, till May in Bovec breed and till December in Istrian breed. Ovarian activity in Jezersko-Solchava ewes lasted 2 to 6.5 months (average 3.86 ± 0.58 months) and in Bovec ewes from 1,5 to 7,5 months (averagely 3.9 5 ± 0.56 months). In Istrian breed, only two ewes shoved signs of ovarian activity, which lasted 30 or 90 days.

### Serum cortisol

**Figure 1:**
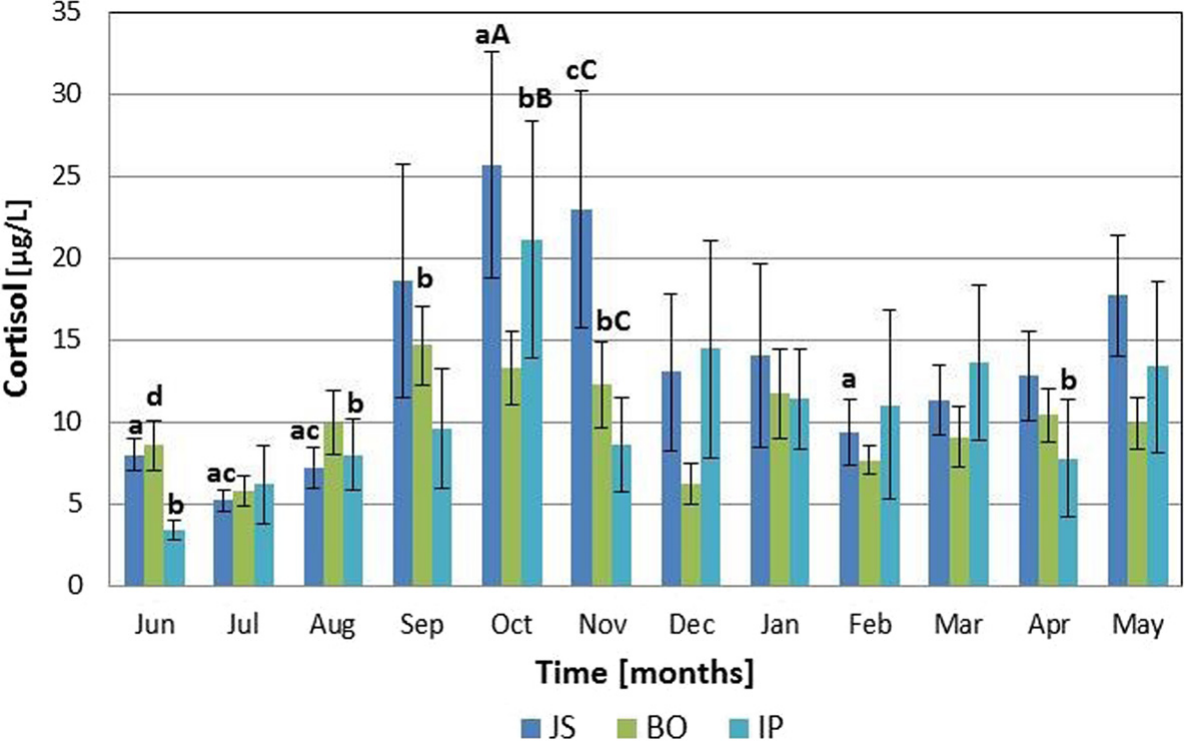
**Monthly cortisol fluctuations in Jezersko-Solchava(JS),Bovec(BO)and Istrian(IP)ewes(mean** **±** **s.e.m.).***Legend*: *a* – *P* <*0.001 for October versus June*, *July*, *August and February* (*in JS*); *b* – *P* <*0.001 for October versus June*, *August*, *November and April* (*in IP*), *c* – *P* <*0.001 for November versus August* (*in JS*), *d* – *P* <*0.05 for September versus June and December* (*in BO*), *A* – *P* <*0.05 for JS versus BO* (*in October*), *B* – *P* <*0.05 for IP versus BO* (*in October*), *C* – *P* <*0.05 for JS versus IP* (*in November*).

Monthly variations in cortisol profiles were recorded in all examined sheep breeds (Figure 1) with the lowest serum cortisol concentrations measured in June for Istrian breed (3.37 ± 0.57 μg/L) and in July for Jezersko-Solchava and Bovec breeds (5.19 ± 0.65 μg/L and 5.76 ± 0.90 μg/L respectively). Thereafter, the cortisol concentration in all breeds increased gradually to reach a significant peak (P <0.001) in September in Bovec (14.67 ± 2.43 μg/L), and in October in Jezersko-Solchava and Istrian breeds (25.69 ± 6.89 μg/L and 21.11 ± 7.25 μg/L). In the following months of the study, the cortisol concentrations decreased again. The RM ANOVA demonstrated significant differences between the monthly cortisol concentration for each breed (P <0.001), but there was no significant interaction (P = 0.239) between each breed and month of sampling. Differences in cortisol concentration between breeds were insignificant for every month of the study with the exception of November between Jezersko-Solchava and Istrian breed (P <0.05).

### Serum insulin

**Figure 2:**
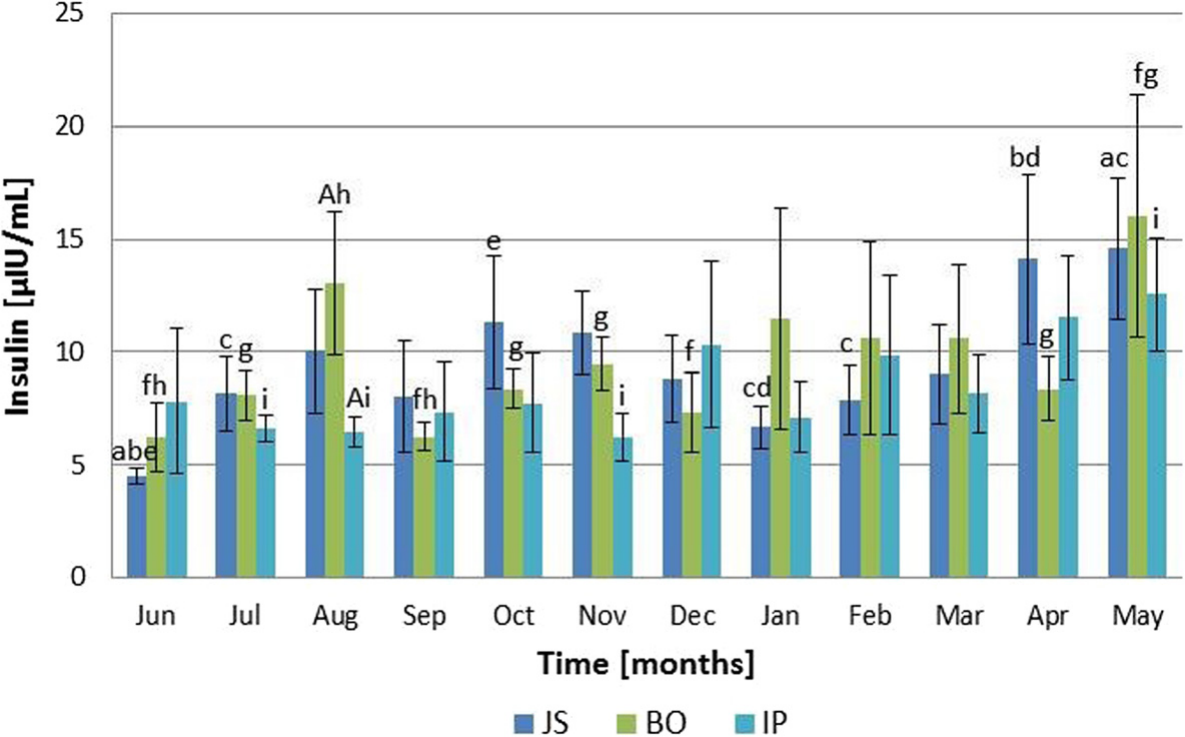
**Monthly insulin fluctuations in Jezersko**-**Solchava(JS),Bovec(BO)and Istrian(IP)ewes(mean** **±** **s.e.m.).***Legend*: *a* – *P* <*0.01 for May vs. June* (*in JS*); *b* – *P* <*0.01 for April vs. June* (*in JS*); *c* – *P* <*0.05 for May vs. July*, *January and February* (*in JS*); *d* – *P* <*0.05 for April vs. January* (*in JS*); *e* – *P* <*0.05 for October vs. January* (*in JS*); *f* – *P* <*0.01 for May vs. June*, *September and December* (*in BO*); *g* – *P* <*0.05 for May vs. April*, *July*, *October and November* (*in BO*); *h* – *P* <*0.05 for August vs. June and September* (*in BO*); *i* – *P* <*0.05 for May vs. July*, *August and November* (*in IP*); *A* – *P* <*0.05in BO versus IP*.

Monthly variations of insulin concentrations were recorded in all sheep breeds included in the study (Figure 2). The lowest serum insulin concentrations were measured in June for Jezersko-Solchava and Bovec breeds (4.53 ± 0.35 μIU/mL and 6.25 ± 1.54 μIU/mL, respectively) and in August for Istrian breed (6.49 ± 0.66 μIU/mL) and the highest in May for Jezersko-Solchava (14.60 ± 3.15 μIU/mL), Bovec (16.03 ± 5.35 μIU/mL) and Istrian breed (12.56 ± 2.52 μIU/mL). The RM ANOVA demonstrated significant differences between the monthly insulin concentration for each breed of ewes (P <0.01), but there was no significant interaction (P = 0.751) between each breed and month of sampling. Differences in insulin concentration between breeds were insignificant for every month of study with the exception of August between Bovec and Istrian breeds (P <0.05).

### Serum NEFA

**Figure 3:**
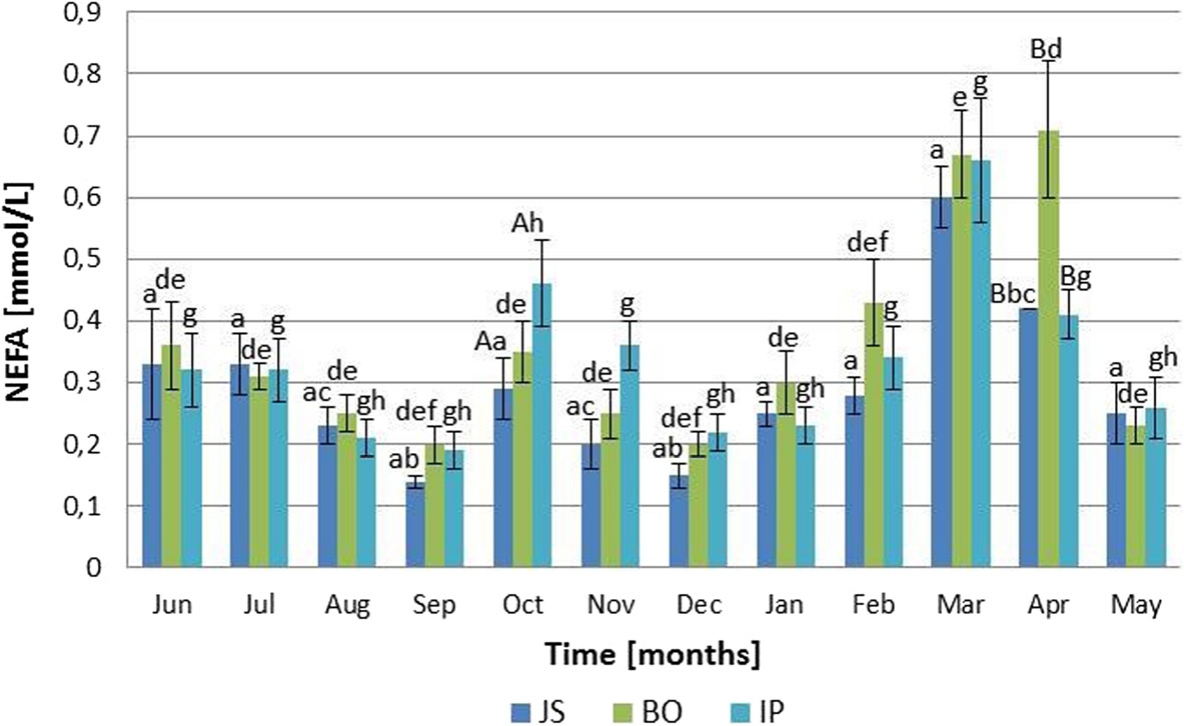
**Monthly NEFA fluctuations in Jezersko**-**Solchava(JS),Bovec(BO)and Istrian(IP)ewes(mean ± s.e.m.).***a* – *P* <*0.001 for March vs. June* – *February and May* (*in JS*); *b* – *P* <*0.001 for April vs. September and December* (*in JS*); *c* – *P* <*0.01 for April vs. August and November* (*in JS*); *d* – *P* <*0.001 for April vs. June* – *February*, *May* (*in BO*); *e* – *P* <*0.001 for March vs. June* – *February*, *May* (*in BO*); *f* – *P* <*0.001 for February vs. September and December* (*in BO*); *g* – *P* <*0.001 for March vs. June* – *September*, *November* – *February*, *April and May* (*in IP*); *h* – *P* <*0.001 for October vs. August*, *September*, *December*, *January and May* (*in IP*); *A* – *P* < *0.01 for IP vs. JS in October*, *B* – *P* < *0.01 for BO vs. JS and IP in April*.

Significant monthly variations in NEFA profiles (Figure 3) were recorded for each of examined sheep breeds, confirmed by the RM ANOVA (P <0.05). The lowest serum NEFA concentrations were measured in September (0.14 ± 0.01 μIU/mL for Jezersko-Solchava, 0.19 ± 0.03 μIU/mL for Bovec and 0.16 ± 0.04 μIU/mL in Istrian breed) while the highest values for Jezersko-Solchava and Istrian breeds were measured in March (0.60 ± 0.05 mmol/L and 0.66 ± 0.10 mmol/L, respectively) and for Bovec breed in April (0.71 ± 0.11 mmol/L). There was no significant interaction (P = 0.144) between each breed and month of sampling. Differences in NEFA concentration between breeds were insignificant for every month of study with the exception of October between Jezersko-Solchava and Istrian breed (P <0.05) and in April for Bovec versus Jezersko-Solchava and Istrian breed (P <0.05).

### Correlations

Correlation coefficients (r^2^) between body weight, serum cortisol, insulin and NEFA concentrations in Jezersko-Solchava, Bovec and Istrian ewes are represented in Table 3.

**Table 3:** **Correlation coefficients** (**r**^**2**^) **between the body weights** (**BW**), **serum cortisol**, **insulin**, **and NEFA concentrations in Jezesko**-**Solchava** (**JS**), **Bovec** (**BO**) **and Istrian** (**IP**) **breeds of sheep**

		**BW**	**cortisol**	**insulin**	**NEFA**
JS (n = 10)	BW	1	0.260	0.632*	0.432
	cortisol	0.260	1	0.434	-0.209
	insulin	0.632*	0.434	1	0.197
	NEFA	0.247	0.542	-0.048	1
BO (n = 10)	BW	1	-0.012	0.413	0.394
	cortisol	-0.012	1	0.005	-0.109
	insulin	0.413	0.005	1	0.080
	NEFA	0.394	-0.109	0.080	1
IP (n = 10)	BW	1	0.490	0.742**	-0.006
	cortisol	0.490	1	0.254	0.308
	insulin	0.742**	0.254	1	-0.351
	NEFA	-0.006	0.308	-0.351	1

Legend: *significant correlation (P <0.05), **significant correlation (P <0.01).

## Discussion

All measured parameters had shown fluctuations over the observation period (Figures 1, 2 and 3). In contrast to our expectations, there were no significant differences between the breeds when comparing monthly values of the measured parameters.

Cortisol levels measured at all sampling times and in all examined breeds were in the range determined in some other sheep breeds, like in Rommey Marsh ewes (5 – 20 μg/L) [24], Dorset-sired crossbred ewes (10 – 20 μg/L) [11] and mature Merino ewes (below 20 μg/L) [25], but lower than in Comisana ewes (30 – 40 μg/L) [9] or Suffolk × Hampshire ewes (28 – 38 μg/L) [10], all of them being tethered or housed in pens.

Cortisol has shown a similar pattern of fluctuations in all examined breeds (Figure 1) and insignificant between-breed differences, with the lowest values observed during summer, intermediate in spring and winter and the highest values being observed in autumn. We believe that stress situations like relocation of sheep to a foreign environment or isolation from the flock, immunological or psychological stressor [7-11] were not reasons for the observed cortisol fluctuations, as the investigated ewes were familiar with humans, used to various procedures, not separated from each other and treated carefully during samplings to avoid any excitement or escaping. Another argument for our conclusion are cortisol concentrations, which were reported for sheep subjected to various forms of stress, exceeding the values obtained in our presented study (see Figure 1). Isolation stress in Comissana [9] and in Dorest-sired crossbred ewes [11] induced the rise of cortisol concentrations (over 90 μg/L and 120 μg/L respectively), while shearing and yarding of mature Merino ewes increased cortisol concentrations to the values over 60 μg/L [25]. Elevation of cortisol concentrations was also measured after the ACTH administration (over 46 μg/L in Suffolk × Hampshire ewes [10], over 80 μg/L in Dorest-sired crossbred ewes [11]). High levels of cortisol in autumn and winter have also been found in other sheep breeds [3,6]. A vital factor that stimulates the pituitary-adrenal axis and cortisol secretion in autumn and winter months might be increased production of LH, promoting the onset of puberty [26] and seasonal reproductive activity in yearling ewes, which started between September and November, as is seen from the estimation of ovarian activity (Table 2). Therefore, a factor that probably contributed to observed cortisol elevations in autumn months was the onset of puberty in the ewes of all three examined breeds. Another explanation for cortisol levels peaking in autumn and not in winter might be associated with decreased environmental temperatures in autumn [18], presenting a strong stress factor for young ewes [27], which have not yet been protected with dense and long wool at that period [28]. An increase of blood cortisol was reported in sheep during long-term cold exposure [29,30], followed by a metabolic acclimatization [30]. In the present study, cortisol levels in September and October were almost the same as were found in the study of Graham et al. [30] who determined cortisol in sheep during cold exposure. On the other hand, low cortisol concentrations during the summer were probably associated with high environmental temperatures; this phenomenon has also been found in heifers [31]. Thus, results of this study suggest that increased cortisol secretion was associated with low temperatures and reproduction activity or combination of both. However, in September and October reproduction activity was found only in some particular animals (Table 2), therefore, we believe that decrease of environmental temperature at that time leads to stimulation of cortisol secretion.

Insulin levels in all three breeds at all sampling times were in line with insulin values reported for adult Romane breed ewes [32] reared outdoors (10 – 30 μIU/mL) and for individually housed Merino lambs [13] of both genders aged 60 and 360 days (5 μμU/mL and 5 – 10 μμU/mL, respectively) but higher than in individually housed Soay rams [14] during short-day or long day photoperiod (0.17 ± 0.05 μμU/mL and 0.16 ± 0.01 μμU/mL). Several other studies report insulin concentrations in various breeds of sheep, like in pregnant blue-faced Leichester cross Swaledal ewes [33], western White-Faced ewes [34] and Rambouillet ewe lambs [35], kept indoors in individual pens. The sheep in these studies [33-35] were kept under various experimental conditions, various procedures were used for insulin measurement and the results were presented in other units, which all make these results difficult to compare with results of our study.

In Jezersko-Solchava breed mean annual insulin and cortisol values were both found to be high, followed by the values determined in Bovec and Istrian breeds, however the differences were insignificant. In evaluating the results of our study, we believe that some metabolic parameters (e. g. blood glucose level) are kept by the maintenance of relations between blood insulin and cortisol concentrations due to their physiological antagonistic effect. Similarly to Gatford et al. [13], a gradual increase of insulin levels was found in the examined ewes, which was in parallel to their growth. Additionally, significant positive correlation between body weight and insulin level was found in Jezersko-Solchava and Istrian breeds (Table 3). It is reported that sensitivity to insulin in humans decreases during and after puberty [36]. Thus, as body weight increases, higher insulin secretion is required to reach the same effect as was reached with lower levels in younger animals. The results of the presented study indicate a similar effect in investigated sheep breeds.

In contrast to insulin, NEFA concentrations were found to significantly fluctuate over the year (Figure 3). NEFA values in all three examined breeds were found to be low during most of the year and were comparable to other sheep breeds with positive energy balance [14,32].

NEFA concentrations in adult Romane breed ewes reared outdoors ranged from approximately 0,9 mmol/L before to 0.2 mmol/L after weaning and increased again to the values of about 0.6 mmol/L after mating, reflecting various levels of body reserve mobilisation harmonized with energy needs in various states of reproductive activity [32]. In adult Soay rams an increase of NEFA levels was observed [14] after the 60-day period of restricted feeding, which has followed a period with freely available food (0.285 ± 0.047 vs. 0.461 ± 0.056 mmol/L in short-day photoperiod and 0.106 ± 0.012 vs. 0.310 ± 0.031 mmol/L long-day photoperiod). In the present study NEFA levels peaked in March (Jezersko-Solchava and Istrian breeds) or April (Bovec breed). Another NEFA peak was detected in October in all examined breeds, significant only in Istrian breed. Blood NEFA concentrations were found to be elevated during times of insufficient feed supply, since low blood glucose levels induce the mobilization of fatty acids from adipose tissue in the form of NEFA [15 – 17]. Additionally, a low insulin level is also significant factor that stimulates the hormone-sensitive lipase activity, resulting in NEFA mobilization [37,38]. Therefore, elevated NEFA values measured in the investigated ewes can be considered as markers for insufficient feed supply in October, and more prominent in March and April. Slow growth in Bovec and Istrian ewes and even loss of body weight in Jezersko-Solchava ewes were found during the same period (Table 1), which also indicates the inadequate supply with nutrients. Losses of dry matter and nutrients in large round bales, stored outside, as also at our farm, ranges from 3 to 40%, with increase of fibre concentrations due to the loss of non-fibre constituents. Because the loss is primarily highly digestible nutrients, the digestibility of forage dry matter decreases during storage [39], which can also be the reason for decreased quality of the food, available to investigated ewes in spring months.

Similarly, increased NEFA levels in October could be a result of low herbage quality in autumn and, therefore, qualitatively insufficient feed supply. The studies of feed in dry Karst pastures have shown the reduction of herbage quality in summer and autumn months, which is caused by drought, which is a recurring problem in the dry Mediterranean karst grasslands [40,41]. This presumption was confirmed by similar NEFA fluctuations in all three tested breeds, which were bred under the same conditions.

Nevertheless, the results of the present study do not differentiate between endogenous (e.g. growth, onset of puberty, circannual hormone patterns, etc.) and exogenous (e.g. feed supply, environmental conditions, etc.) factors that influence monthly fluctuations of hormone secretion and energy metabolism. We believe that yearling ewes of various breeds bred across the Northern Mediterranean are subjected to similar environmental factors during their growth, since they are born in the late winter months. Considering this, cortisol, insulin and NEFA annual fluctuations in growing ewes could be somewhat universal and present additional data in understanding the regulation of energy metabolism and the adaptability to environmental conditions in the studied category of sheep.

## Conclusions

We conclude that in the Jezersko-Solchava, Bovec and Istrian breeds of sheep, the annual fluctuations of cortisol, insulin and NEFA were present, following the similar patterns in all examined breeds. Between-breed differences in monthly values of examined parameters were insignificant. Elevated cortisol levels were measured in autumn and could be associated with the onset of puberty and seasonal reproductive activity and/or low environmental temperature as a stress factor. Insulin levels in growing ewes increased with body weight and were probably not significantly influenced by environmental factors. Elevated NEFA levels were found in autumn and spring, corresponding to an insufficient quality of feed supply that was available to sheep. Nevertheless, the evaluation of annual cortisol, insulin and NEFA fluctuations presents further information in the understanding of energy metabolism regulation and adaptive mechanisms to environmental conditions in growing ewes.

## Abbreviations

NEFA: Non-esterified fatty acids; EIA: Enzyme immunoassay; CV: Coefficient of variation; RM ANOVA: Repeated measures analysis of variance; BW: Body weight; LH: Luteinizing hormone; ACTH: Adrenocorticotropic hormone.

## Competing interests

The authors declare that they have no competing interests.

## Authors’ contributions

TS has actively participated in both data analyses and preparation of the manuscript; ZJ has organized the samplings, performed statistical analyses and collaborated in preparation of the manuscript; NCK was the leader of the project and has performed the sample analyses as well as collaborated in preparation of the manuscript. All authors read and approved the final manuscript.
